# Epigenetic alterations in patients with anorexia nervosa—a systematic review

**DOI:** 10.1038/s41380-024-02601-w

**Published:** 2024-06-07

**Authors:** Larissa Käver, Anke Hinney, Luisa Sophie Rajcsanyi, Hannah Benedictine Maier, Helge Frieling, Howard Steiger, Clara Voelz, Cordian Beyer, Stefanie Trinh, Jochen Seitz

**Affiliations:** 1 Institute of Neuroanatomy, RWTH Aachen University, Wendlingweg 2, 52074 Aachen, Germany; 2 Department of Child and Adolescent Psychiatry, Psychosomatics and Psychotherapy, University Hospital Essen, Virchowstrasse 174, 45147 Essen, Germany; 3 Center for Translational and Behavioral Neuroscience, University Hospital Essen, Hufelandstraße 55, 45147 Essen, Germany; 4 Department of Psychiatry, Socialpsychiatry and Psychotherapy, Hannover Medical School, Carl-Neuberg-Str. 1, 30625 Hannover, Germany; 5 Department of Psychiatry, McGill University, Montreal, QC H3A 1A1 Canada; 6 Department of Child and Adolescent Psychiatry, Psychosomatics and Psychotherapy, RWTH Aachen University, Neuenhofer Weg 21, 52074 Aachen, Germany; 7 Department of Child and Adolescent Psychiatry, Psychosomatics and Psychotherapy, LVR University Hospital Essen, Virchowstrasse 174, 45147 Essen, Germany

**Keywords:** Genetics, Neuroscience, Psychology, Psychiatric disorders

## Abstract

Anorexia nervosa (AN) is a complex metabolic and psychological disorder that is influenced by both heritable genetic components and environmental factors. Exposure to various environmental influences can lead to epigenetically induced changes in gene expression. Epigenetic research in AN is still in its infancy, and studies to date are limited in determining clear, valid links to disease onset and progression are limited. Therefore, the aim of this systematic review was to compile and critically evaluate the available results of epigenetic studies specifically in AN and to provide recommendations for future studies. In accordance with the PRISMA guidelines, a systematic literature search was performed in three different databases (PubMed, Embase, and Web of Science) through May 2023. Twenty-three original papers or conference abstracts on epigenetic studies in AN were collected. Epigenome-wide association studies (EWASs), which analyze DNA methylation across the genome in patients with AN and identify potential disease-relevant changes in promoter/regulatory regions of genes, are the most promising for future research. To date, five EWASs on AN have been published, suggesting a potential reversibility of malnutrition-induced epigenetic changes once patients recover. Hence, determining differential DNA methylation levels could serve as a biomarker for disease status or early diagnosis and might be involved in disease progression or chronification. For future research, EWASs with a larger sample size, longitudinal study design and uniform methods should be performed to contribute to the understanding of the pathophysiology of AN, the development of individual interventions and a better prognosis for affected patients.

## Introduction

Anorexia nervosa (AN) is a complex psychiatric disorder characterized by severe restriction of food intake leading to dangerous underweight, intense fear of gaining weight, and a distorted body image [1, 2]. AN has a high clinical relevance, as it usually occurs during adolescence in girls and young women and has a lifetime prevalence of 1–4% in Europe [3–5]. AN has the highest mortality risk among all mental disorders, and 7–10 years after being diagnosed with AN, less than 50% recover completely [1, 6]. Despite extensive research efforts, the underlying mechanisms contributing to the development and maintenance of AN as well as comorbid mental disorders remain largely unclear.

AN is a neuropsychiatric phenotype that has a significant heritable component. Epidemiological studies have shown an 11-fold greater risk of developing this disorder in first-degree relatives of AN patients [7, 8]. Among monozygotic twins (who share 100% of their segregating genes) and dizygotic twins (who share approximately half of their genetic material), heritability estimates range from 48 to 84% [9–11]. This high heritability rate, although still far from 100%, suggests that AN is influenced by a complex pattern of etiology involving both genetic and environmental aspects.

AN has been reconceptualized as a metabo-psychiatric disorder, as for AN significant genetic correlations with several psychiatric disorders, bodyweight and metabolic phenotypes were identified in a comprehensive genome-wide association study (GWAS). This study, encompassing 16,992 patients with AN and 55,525 controls, revealed significant associations with psychiatric conditions such as schizophrenia, and obsessive-compulsive or major depressive disorder, alongside disturbances in glucose and lipid metabolism [12–14].

In recent years, epigenetic mechanisms have become increasingly interesting to researchers, as these mechanisms provide a supplementary level of gene regulatory information, potentially bridging external and internal environmental cues with genetic programming [15–17]. Epigenetic changes are highly relevant to AN as they represent potential mechanisms that connect the genetic predisposition for AN with environmental factors such as stress, nutrition, medication intake or traumatic events. Epigenetics encompass a variety of molecular changes that influence gene activity without altering the DNA sequence itself [18]. From a biological perspective, three groups of epigenetic mechanisms have been described: DNA modifications, histone modifications and non-coding RNAs. These epigenetic changes can lead to long-term alterations in gene expression, thereby increasing or decreasing the risk of developing AN and potentially influencing its course.

The most studied chemical modification of DNA is DNA methylation, which can be quantified by high-throughput microarray techniques such as the Illumina Infinium Human Methylation EPIC Bead Chip Kit, which can simultaneously assess DNA methylation of one million CpG (cytosine-guanine) sites in a single experiment. In DNA methylation, a small methyl group is covalently bound to the 5’ carbon of cytosine in a CpG dinucleotide, where guanine follows cytosine in the 5’ to 3’ direction on one DNA strand [18–20]. For a variety of psychiatric disorders, changes in the methylation pattern along the genome have already been described compared to healthy controls. These include, most notably, major depressive disorder [21, 22], one of the most common comorbidities in AN, and autism spectrum disorder [23, 24].

DNA methylation patterns are relatively stable. However, they can also be reversed by an active demethylation process in which the methyl group is oxidized by ten-eleven-translocation enzymes (TETs), leading first to hydroxymethylcytosine (5hmC), followed by formylcytosine (5fC) and then carboxycytosine (5caC) [25–27]. The stable modification 5hmC is of special interest in the context of neuropsychiatric disorders because the highest concentrations can be identified in neurons of the brain [27–30]. The second mechanism, histone modifications, involves methylation, acetylation or phosphorylation of amino acids in histone proteins that affect gene regulation due to chromatin accessibility [31]. The third mechanism, non-coding RNA fragments that do not code for proteins themselves, can affect gene regulation by binding to transcription factors, thereby inhibiting gene translation to proteins (posttranscriptional silencing) or by guiding the positioning of nucleosomes along the genome and thereby altering DNA accessibility [32–35].

Epigenetic processes are dynamically influenced by environmental stimuli, such as smoking, stress, nutritional status, or medication intake, and are highly cell specific. Epigenetic patterns cannot be assessed in the brains of living beings [36], but peripheral DNA methylation has been validated as being reflective of brain processes and can thus be informative in a mental-health context [37–40]. Epigenetic profiles, which are often studied in epigenome-wide association studies (EWASs), can be described as biological traits that may be influenced by different disruptive factors (age, smoking, drugs). This means that we need to be aware of the potential confounders that might affect the results when designing experiments and analyzing statistical data [41, 42].

The disease-specific characteristics of AN, including its higher prevalence in females (male-to-female ratio of 1:8) [43], higher vulnerability for disease onset during adolescence and early adulthood [44], and especially the described concordance but also discordance for AN between monozygotic twin pairs (MZ) [45–47], indicate a possible influence of epigenetic processes in the development and progression of this psychiatric disease [13].

The rapid progress in genetic and epigenetic research, facilitated by innovative techniques such as array-based genome-wide analysis, has significantly extended our comprehension of the underlying biology of AN and may lead to the discovery of more effective treatment approaches [48]. The previous systematic review of Hübel et al [13] focused on epigenetic changes in eating disorders in general (anorexia nervosa, bulimia nervosa, binge-eating disorder) and included 13 studies on epigenetics in AN. Their systematic research yielded an inconclusive picture of the existing results based on the available data, with most of the reviewed studies including small case numbers and only two EWASs delivering the first pilot results [13, 45, 49]. Other non-systematic reviews also focused on eating disorders in general but concentrated more on the most promising EWAS results or summarized genetic and epigenetic aspects generally [40, 50, 51]. As the body of literature has substantially grown since the last systematic review, which covered studies until 2017, the primary objective of this systematic review was to synthesize and critically evaluate the newly available studies investigating epigenetic alterations in individuals with AN. By extending the search period until 2023, it was possible to include twice as many studies and especially to focus on the analysis of epigenetic changes on an epigenome-wide level (EWASs). By systematically analyzing and summarizing the findings from past and recent studies, we aimed to provide a comprehensive overview of the epigenetic changes observed in AN and identify common patterns or alterations across different types of epigenetic modifications. Furthermore, we will discuss limitations and provide insights and recommendations for future investigations. A better understanding of epigenetic alterations that are associated with AN could lead to the development of targeted therapeutic interventions and improved clinical management strategies for individuals affected by this severe eating disorder.

## Methods

### Search strategy

**Fig. 1:**
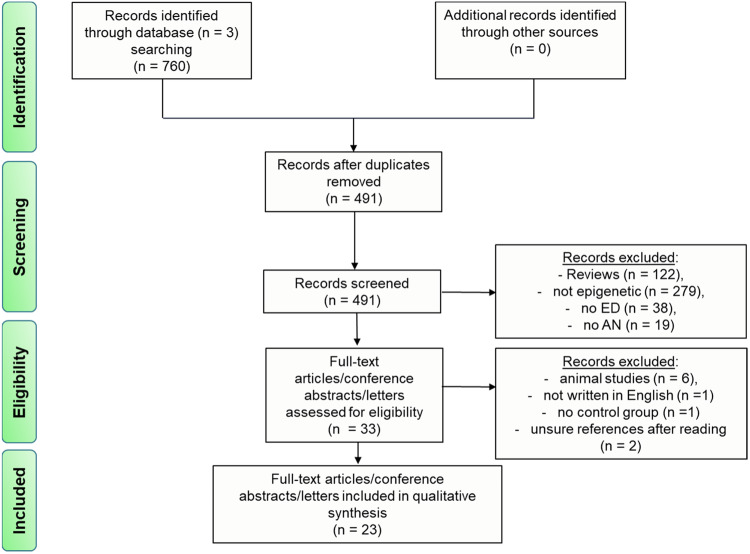
PRISMA flow diagram of selected and screened references. ED eating disorder, AN anorexia nervosa.

The present systematic review was designed in accordance with the PRISMA guidelines [52]. We conducted an extensive systematic literature search from May 04, 2023, to June 01, 2023, in three different scientific literature databases (PubMed, Web of Science, Embase) with no time limitation. The first published article on epigenetics in the context of eating disorders appeared in January 2003 and therefore symbolizes the starting point of the present systematic literature search. Our search strategy in all three databases used key search terms including (#1 anorexia OR anorexia nervosa OR eating disorder) AND (#2 epigenetic OR epigenetics OR methylation OR histone OR non-coding RNA). We selected all publications found according to the previously defined inclusion/exclusion criteria (see Section “Selection criteria”) and afterward screened these references for eligibility. In accordance with the PRISMA guidelines, Fig. 1 displays our search results and the corresponding selection process in a PRISMA flow diagram.

### Selection criteria

We included studies that met the following criteria:

- Publication in peer-reviewed English or German journals
- Original articles
- Cross-sectional and longitudinal studies
- Human studies
- Clinical diagnosis of AN according to Diagnostic and Statistical Manual of Mental Disorders (DSM) versions IV or 5
- Investigation of any type of epigenetic mechanism: methylation, histone modification, non-coding RNAs
- Inclusion of a control group or a longitudinal within-participant comparison

Publications that were identified by the systematic literature search strategy were screened by one author (LK) in a two-step process. First, the titles, abstracts, and key words of each publication were reviewed to exclude references that were clearly noneligible studies. Then, the remaining full-text articles were reviewed against the selection criteria, and information for each publication was extracted to better compare the studies with each other (see Section “Data extraction”).

### Data extraction

We extracted the following data from every identified publication:

- Author
- Publication year
- Sample (including number of cases and controls, sex, mean age, mean BMI, diagnosis)
- Study design (cross-sectional/longitudinal)
- DNA source
- Outcome variables (global DNA methylation level, candidate genes, EWAS, number of CpGs)
- Exclusion criteria
- Limitations

Considering the high heterogeneity of the results obtained in the identified studies, a quantitative synthesis of the data in the form of a meta-analysis was not possible. Instead, a qualitative systematic review was chosen to capture the associations between epigenetic changes and the pathophysiology of AN.

## Results

Overall, 491 publications on epigenetic alterations in anorexia nervosa listed in three electronic databases (PubMed, Embase, Web of Science) until June 2023 were identified after the deletion of duplicates. According to our predetermined selection criteria (see Section “Selection criteria”), 458 of these records were excluded after screening. A total of 122 reviews, 297 studies not including epigenetic processes, 38 studies not related to eating disorders, and 19 studies not related to AN were excluded. This resulted in 33 full-text articles or conference abstracts that were assessed for eligibility. After a second screening process, another six studies were excluded because they investigated animals and not humans, one was not written in English, two were excluded after reading because they did not fit the criteria, and another study did not include a control group. Finally, 23 publications with a total of 1075 patients and 956 healthy controls (overlapping controls were considered only once) were included in the present qualitative synthesis (Fig. 1). To our knowledge, these 23 papers correspond to all available studies that have analyzed epigenetic changes in AN from the first publication in January 2003 until June 2023.

Among these 23 publications, four studies determined the global DNA methylation pattern in patients with AN compared to controls, five studies analyzed the methylation profiles across epigenome-wide association studies (EWASs) using high-throughput microarray techniques, and 16 studies investigated candidate genes in AN. Only two publications used a longitudinal research design [53, 54], while all the others were cross-sectional. One EWAS examined the epigenome of monozygotic twin pairs discordant for AN [47], while another used this study to validate the achieved results [45]. Interestingly, all studies focused exclusively either on adolescent or adult female patients with an age range between 16-60 years and considered only DNA methylation profiles. None of the included studies analyzed other epigenetic processes, such as modifications on histone proteins or non-coding RNAs (Table. 1). Although AN has both psychiatric and metabolic components, all studies measured epigenetic alterations in whole blood, saliva-derived DNA, or buccal cells and not brain or adipose tissue, which is common in human studies (Table. 1). Moreover, some of the publications were based on the same patient data or used an expanded number of participants. Four studies recruited patients from the homocysteine and DNA methylation in eating disorders (HEaD) study [55–58], two used samples from the Charité Berlin in Germany [59, 60] and another four used probands acquired from the Douglas Institute Eating Disorders Program in Montreal, Canada [49, 54, 61, 62]. Therefore, there was some sample overlap. Furthermore, three studies failed to discuss the limitations arising from their experimental design. Characteristics from every included study according to our defined data extraction criteria are summarized in Table. 1.

**Table 1:** A: Overview of included studies for global DNA measurements. B: Overview of included EWASs. C: Overview of included candidate gene studies.

Author (Year)	Sample					Study design	Tissue	Methylation results		Exclusion criteria	Limitations	Comments
	N	Sex	Age (SD) [years]	BMI (SD)	Diagnosis			Global methylation (or genes)	AN (compared to control)			
**A:**												
Booij et al. [49]	AN: n = 29 (restrictive n = 10; binge/purge n = 19) CO: n = 15	100% female	AN restrictive: 21.5 (6.4) AN binge/purge: 23.4 (5.6) CO: 24.2 (5.8)	AN restrictive: 14.9 (1.8) AN binge/purge: 15.9 (1.1) CO: 21.9 (2.1)	DSM-5	Cross-sectional	Leukocytes	**Global DNAm** (14 CpGs) (representing genes thought to be associated with histone acetylation, RNA modification, cholesterol storage and lipid transport, dopamine and glutamate signaling)	↑	CO: past or present psychiatric disorder; psychoactiv medications	All: cross-sectional (unknown whether the observed methylation differences are related to pre-existing characteristics that predispose individuals to AN, or are consequences of a prolonged course of AN)	
Saffrey et al. [64]	AN: n = 10 CO: n = 10	100% female	AN: 21.5 (10.16) CO: 23.1 (9.45)	AN: 16.31 (1.76) CO: 25.43 (5.29)	n.a.	Cross-sectional	Buccal cells	**Global DNAm** **IGF2** (insuline-like growth factor)	↔ ↔	n.a.	All: small n; tissue specificity	
Tremolizzo et al. [63]	AN: n = 32 CO: n = 13	100% female	AN: 15.5 (1.4) CO: 16.3 (1.3)	AN: 15.5 (2.1) CO: ≥21	DSM-IV	Cross-sectional	Blood	**Global DNAm**	↓	All: hematological or hepatic disorders, cancer, recent infections or surgery, age <14 and >18 years, alcohol abuse CO: neurological or psychiatric disorder, medication	n.a.	
Frieling et al. [58]	AN: n = 22 CO: n = 30	100% female	AN: 26.5 (10.3) CO: 22.0 (4.8)	AN: 15.9 (2.0) CO: 21.7 (3.7)	DSM-IV	Cross-sectional	Blood	**Global DNAm** **SNCA** (alpha synuclein gene; thought to play a role in regulating neurotransmitter release in nerve cells) **HERP** (homocysteine-induced endoplasmatic reticulum protein encoding gene; plays a role in maintaining ER homeostasis and response to stressful conditions)	↓ ↑ ↔	n.a.	n.a.	
**B:**												
Steiger et al. [54]	AN active: n = 145, AN remitted: n = 49 (1-year remission; BMI > 18.4 for 12 month) CO: n = 64	100% female	AN (active): 24.73 (7.4) AN (remitted): 27.39 (6.2) CO: 24.5 (5.4)	AN (active): 15.05 (1.8) AN (remitted): 21.86 (2.7) CO: 22.59 (2.6)	DSM-5	Cross-sectional	Blood/leukocytes	NR1H3 (AN-active) Generally: many genes (differentially methylated) for AN (active) vs. CO No differences between AN-remitted vs CO Lower BMI correspond to lower DNAm --> genes: corresponds to stress, leptin receptor function or lipid metabolism	↑ ↓	AN: endocrinologic, diabetic, neoplastic or cardiovascular diseases; substance dependence, psychosis or bipolar illness (BUT: controlled statistically for effects attributable to age, smoking and psychotropic medication)	All: tissue specificity of DNAm; no gene expression measured --> significance of altered methylation not clear	
Iranzo-Tatay et al. [47]	n= monozygotic twins discordant for AN MZ AN: n = 7 MZ CO: n = 7 AN patients: n = 7 CO: n = 7	100% female	MZ AN: 20.42 (7.39) MZ CO: 20.42 (7.39) AN: 20.14 (7.08) CO: 21.42 (7.48)	MZ AN: 16.75 (1.43) MZ CO: 19.85 (1.06) AN: 15.62 (0.95) CO: 19.87 (1.2)	DSM-5	Cross-sectional	Blood	9 CpGs detected (significant altered methylation)	n.a (altered)	AN: family-related to MZ AN CO: psychoactive medications	All: tissue specificity of DNAm (blood and not brain); small n; not longitudinal (for causality of methylome findings) MZ AN/MZ CO: did not control for twin chronicity, shared the same placenta (monochorionic or dichorionic) --> Chronicity is considered to be influential in epigenetic status	
Steiger et al. [61]	AN active: n = 75 (from n = 52 DNAm after intervention; 114 days average of weight restoration), AN remitted: n = 31 (1-year remission; BMI > 18.0 for 12 month) CO: n = 41	100% female	AN (active, restrictive type; n = 34): 24.88 (8.39) AN (active, binge/purge type; n = 41): 23.41 (5.28) AN (remitted): 26.81 (5.41) CO: 23.93 (5.33)	AN (active, restrictive type; n = 34): 14.39 (1.72) AN (active, binge/purge type; n = 41): 15.78 (1.20) AN (remitted): 21.10 (2.02) CO: 22.55 (2.38)	DSM-5	Longitudinal	Blood	Global DNAm 58 CpGs detected corresponding to 55 genes (differentially methylated)	↑ (for 53 of 58 CpGs (91.4%))	AN: endocrinologic, diabetic, neoplastic or cardiovascular diseases; substance dependence, psychosis or bipolar illness	All: tissue specificity; small n; uncontrolled effects of medication and substance use	Follow-up methylation and clinical data on 52 women (28 with anorexia nervosa restrictive type and 24 with anorexia nervosa binge/purge type) after an average of 114 of treatment emphasizing weight restoration
Kesselmeier et al. [45]	AN: n = 47 1.CO (lean without AN): n = 47 2.CO (population-based): n = 100	100% female	AN: 16 (n.a.) lean: 22 (n.a.) CO (population-based): 60 (n.a.)	AN: 13.7 (n.a.) lean: 17.3 (n.a.) CO (population-based): 26.6 (n.a.)	DSM-IV	Cross-sectional	Blood	Global DNAm AN vs. Lean: 51 CpGs (differentially methylated) AN vs population-based: 81 CpGs (differentially methylated) TNXB	↑ (compared to both control groups)	lean: somatic disorder; >10 cigarettes per day; life-time occurrence of AN and bulimia nervosa	All: no influence of BMI/hormone effect or smoking tested; small n for high-throughput DNAm; blood instead of brain tissue AN: no particular sub-phenotypes or more quantitative endophenotype comparisons CO (population-based): age much more higher than AN or lean (e of the potentially differentially methylated CpG sites between AN and POP might be confounded by age)	Validated by twin study (discordant for AN)
Booij et al. [49]	AN: n = 29 (restrictive n = 10; binge/purge n = 19) CO: n = 15	100% female	AN restrictive: 21.5 (6.4) AN binge/purge: 23.4 (5.6) CO: 24.2 (5.8)	AN restrictive: 14.9 (1.8) AN binge/purge: 15.9 (1.1) CO: 21.9 (2.1)	DSM-5	Cross-sectional	Leukocytes	Global DNAm (14 CpGs) (representing genes thought to be associated with histone acetylation, RNA modification, cholesterol storage and lipid transport, dopamine and glutamate signaling)	↑	CO: past or present psychiatric disorder; psychoactive medications	All: cross-sectional (unknown whether the observed methylation differences are related to pre-existing characteristics that predispose individuals to AN, or are consequences of a prolonged course of AN)	
**C:**												
He et al. [67]	AN: 91 CO: 87	100% female	AN: 18.96 (4.14) CO: 19.95 (3.74)	AN: 15.22 (2.08) CO: 20.02 (3.20)	DSM-5	Cross-sectional	Blood	SLC6A4	↑	All: medication, organic or psychiatric disorder; smoking, drinking, drugs CO: score <20 (EAT 26); <19 (BDI-II); <45 (BAI)	All: not perform multiple testing for corrections CO: no effects of malnutrition on SLC6A4 methylation; environmental factors and patients’ nutritional level not included	
Franzgo et al. [66]	AN restrictive (AN0): n = 45, AN purging (AN1): n = 21 CO: n = 34	100% female	AN0: 16.0 (n.a.) AN1: 18.0 (n.a.) CO: 25.0 (n.a.)	AN0: 14.4 (n.a.) AN1: 15.8 (n.a.) CO: 20.5 (n.a.)	DSM-5	Cross-sectional	Blood	SLC6A4	↓ (for AN0)	All: mental retardation, dementia, schizophrenia, Turner’s syndrome, other neurological disorders, underlying endocrine pathologies CO: ever diagnosed with an eating disorder	CO: small sample size	
Batury et al. [70]	acutely underweight AN (acAN): n = 39, AN recovered (recAN): n = 22 (BMI > 18,5 (if older than 18years) or BMI>10th BMI percentile (if younger than 18 years) for 3 months) CO: n = 54	100% female	acAN: 18.0 (3.4) recAN: 19.7 (3.8) CO: 17.5 (2.8)	acAN: 15.0 (0.3) recAN: 21.3 (0.4) CO: 21.4 (0.3)	DSM-5	Cross-sectional	Blood	LEPR (appetite regulation) GHSR1a (appetite regulation) (compared to recAN and HC)	↔ (adolescents) ↑ (“ghrelin-resistance” hypothesis in AN)	All: IQ < 85, current inflammatory, neurological or metabolic illness, chronic bowel diseases, cancer anemia, pregnancy, breast feeding, treatment with cortisone or psychotropic medications within past 6 weeks, drug consumption AN: organic brain syndrome, schizophrenia, substance dependence, bipolar illness, bulimia nervosa, binge-eating disorder CO: psychiatric illness	AN: specific for very young patients; not longitudinal; tissue specificity; average of CpG methylation across promotor, not individual CpGs	
Boehm et al. [38]	AN: 55 CO: 55	100% female	AN: 16.15 (3.07) CO: 16.17 (3.07)	AN: 14.55 (1.35) CO: 20.68 (2.37)	DSM-5	Cross-sectional	Blood, fMRI for resting-state functional connectivity (rsFC)	SLC6A4	AN: methylation correlated positively with rsFC in right dorsolateral cortex ( ↑ methylation = ↑ rsFC) CO: methylation correlated negatively with rsFC in right dorsolateral cortex ( ↑ methylation = ↓ rsFC)	All: psychotropic medication (other than selective serotonin reuptake inhibitors (SSRIs)) within 4 weeks before study (n = 1), binge eating, bulimia nervosa, substance buse, neurologic or medical conditions	All: not longitudinal, small n, tissue specificity, no genetic variability and other influencing factors such as stress and childhood adversity	
Thaler et al. [62]	AN active: n = 69, AN remitted: n = 21 (1-year remission; BMI > 18.0 for 12 month) CO: n = 35	100% female	AN (active): 23.53 (5.78) AN (remitted): 27.43 (5.54) CO: 23.83 (5.29)	AN (active): 15.21 (1.65) AN (remitted): 21.52 (2.05) CO: 22.51 (2.49)	DSM-5	Cross-sectional	Blood/leukocytes	OXTR	↑ (AN-active compared to AN-rem and HC)	CO: history of ED behavior or diagnosis; BMI between 18.0-30.0; current psychological diagnosis; psychiatric medication	All: tissue specificity of DNAm; lack of control gene (cannot conclude that the effects are associated with epigenetic effects owing solely to OXTR, rather than genomewide influences); small n; did not control for cigarette smoking in the samples which has been shown to affect DNA methylation	
Neyazi et al. [53]	AN (LEP gene): n = 129 CO (LEP gene): n = 117 AN (LEPR, Leptin receptor): n = 135 CO (LEPR): n = 119	100% female	AN (LEP): 27.31 (7.6) CO (LEP): 29.47 (9.3) AN/CO (LEPR): n.a.	AN (LEP): T0: 16.8 (0.94) T1: 17.6 (1.72) T2: 18.19 (2.03) CO (LEP): 21.7 (1.8)	DSM-5	Longitudinal (3 timepoints: baseline =T0, end of therapy (40 weeks = T1), 12-months follow-up = T2)	Blood	LEP/LEPR (T0, acute AN) --> patients with lowest LEP DNAm (high leptin expression = no appetite and low food intake) were the patients that fully responded to therapy and fully recovered	↓ (adult patients)	AN: • Current substance abuse • Current medication with neuroleptics • Current suicidal ideation • Psychotic disorder • Bipolar disorder • Serious unstable medical problems/complications • Ongoing psychotherapy • Pregnancy • Clinically relevant cardiac arrhythmia • Primary somatic diseas CO: BMI ≤ 19 or ≥25, eating disorder, other psychiatric disorders, pregnancy, current somatic disorder	All: tissue specificity of DNAm; PCR bias	
Subramanian et al. [71]	AN: n = 28 CO: n = 27	100% female	AN: 24 (6.1) CO: 26 (6.3)	AN: 16 (1.9) CO: 25 (4.5)	DSM-IV	Cross-sectional	Saliva-derived DNA	3 CpGs associated with AN in **HDAC4** one adjacent CpG to Booij et al. [49] 2 adjacent CpGs to Booij et al. [49]	↑ (9%) ↓ (3% and 5%)	CO: history of ED; psychiatric medications at time of saliva-collection	All: tissue specificity; cross-sectional; did not screen all CpGs (522 missing) in HDAC4 gene (352780 bp); no data on variables influencing epigenetics (smoking, non-psychiatric medications, substance use, oral contraception changing estrogen level and therefore HDAC4 expression); no mRNA expression measured because only DNA and no tissue samples	
Kim et al. [69]	AN: n = 15 CO: n = 36	100% female	AN: 24.7 (10.7) CO: 22.1 (2.2)	AN: 15.06 (2.58) CO: 21.04 (2.23)	DSM-IV	Cross-sectional	Buccal cells	**OXTR**	↑ (5 of 6 significant CpGs)	All: smoking, non-heterosexual, parous, medications, including contraceptives, history of psychiatric illness, <18 years old AN: active substance use disorder, psychotic disorder (schizophrenia, schizoaffective, psychosis not otherwise specified), autism, Asperger’s syndrome	All: small n; cross-sectional; tissue specificity; did not assess smoking behavior or folate levels	
Saffrey et al. [64]	AN: n = 10 CO: n = 10	100% female	AN: 21.5 (10.16) CO: 23.1 (9.45)	AN: 16.31 (1.76) CO: 25.43 (5.29)	n.a.	Cross-sectional	Buccal cells	Global DNAm IGF2 (insulin-like growth factor)	↔ ↔	n.a.	All: small n; tissue specificity	
Pjetri et al. [68]	AN: n = 45 CO: n = 45	n.a.	AN: 16-60 CO: n.a.	AN: 19.5 (0.44) CO: 22.5 (0.43)	DSM-IV-R	Cross-sectional	Blood	DRD2 (dopamine receptor) LEP BDNF SLC6A4	↔ ↔ ↔ ↔	n.a.	All: tissue specificity AN: relatively high BMI (not acutely ill); randomly selected from a database	
Ehrlich et al. [60]	AN (acute): n = 40 AN (recovered): n = 21 CO: n = 54	100% female	AN (acute): 17.88 (3.23) AN (recovered): 19.25 (3.67) CO: 17.10 (2.29)	AN (acute): 14.98 (1.20) AN (recovered): 20.93 (2.32) CO: 21.39 (2.04)	DSM-IV	Cross-sectional	Blood	POMC	↔	All: IQ < 85, current inflammatory, neurological or metabolic illness, chronic bowel diseases, cancer, anemia, pregnancy, breastfeeding, treatment with cortisone, and use of psychotropic medications within the past 6 months AN: organic brain syndrome, schizophrenia, substance dependence, bipolar disorder, bulimia nervosa, binge-eating disorder	All: small n; did not directly assess folate deficiency; tissue specificity; cross-sectional design	Malnutrition was characterized by plasma leptin; smoking associated with DNAm of POMC gene promotor
Ehrlich et al. [59]	AN (acute): n = 31 AN (recovered): n = 30 CO: n = 30	100% female	AN (acute): 16.4 (1.3) AN (recovered): 19.3 (3.0) CO: 16.4 (1.5)	AN (acute): 15.0 (1.1) AN (recovered): 20.7 (2.2) CO: 20.8 (2.0)	DSM-IV	Cross-sectional	Blood	POMC	↔	All: IQ less than 85, current inflammatory; neurological or metabolic illness; chronic bowel diseases; cancer; anemia; pregnancy; breastfeeding, treatment with cortisone; psychotropic medications within the past 6 months AN-Rec: BMI < 18.5 (if older than 18 years) or a BMI <10th BMI percentile last 3 months prior to study, binged, purged or engaged in significant restrictive eating patterns CO: psychiatric illness, organic brain syndrome, schizophrenia, substance dependence, bipolar illness, bulimia nervosa, binge-eating disorder	All: small n; tissue specificity; cross-sectional design	
Frieling et al. [57]	AN: n = 22 CO: n = 30	100% female	AN: 26.5 (10.3) CO: 22.0 (4.5)	AN: 15.9 (2.0) CO: 21.7 (3.7)	DSM-IV	Cross-sectional	Blood	SLC6A3/DAT (dopamine transporter gene) DRD2 (dopamine receptor 2) DRD4 (dopamine receptor 4)	↑ ↑ ↔	All: high coffee consumption, alcohol abuse, medication, endocrinological conditions, other diseases (i.e., thromboembolic, diabetes mellitus, cardiovascular diseases) CO: any medical or psychiatric condition	All: cross-sectional design, tissue specificity	
Frieling et al. [55]	AN: n = 20 CO: n = 26	100% female	AN: 26.4 (10.6) CO: 21.3 (2.2)	AN: 15.9 (2.0) CO: 20.9 (1.6)	DSM-IV	Cross-sectional	Blood	CNR1/CB1 (cannabinoid receptor1)	↔ (BUT: significantly higher levels of CB1 receptor mRNA in the blood in AN compared to HC)	n.a.	n.a.	
Frieling et al. [56]	AN: n = 22 CO: n = 30	100% female	AN: 22 (18–51) CO: 21 (19–43)	AN: 15.9 (2.0) CO: 20.3 (4.1)	DSM-IV	Cross-sectional	Blood	ANP (atrial natriuretic peptide) Vasopressin	↔ ↔	n.a.	All: small n; tissue specificity	
Frieling et al. [58]	AN: n = 22 CO: n = 30	100% female	AN: 26.5 (10.3) CO: 22.0 (4.8)	AN: 15.9 (2.0) CO: 21.7 (3.7)	DSM-IV	Cross-sectional	Blood	global DNAm SNCA (alpha synuclein gene; thought to play a role in regulating neurotransmitter release in nerve cells) HERP (homocysteine-induced endoplasmatic reticulum protein encoding gene; play a role in maintaining ER homeostasis and response to stressful conditions)	↓ ↑ ↔	n.a.	n.a.	

↑ hypermethylation, ↓ hypomethylation, ↔ no differences in methylation pattern, *AN* anorexia nervosa, *acAN* active anorexia nervosa, *recAN* recovered from anorexia nervosa, *CO* controls, *n/N* number, *SD* standard deviation, *BMI* body mass index, *DSM* Diagnostic and Statistical Manual of Mental Disorders, *n.a.* not available, *CpG* cytosine-phosphate-guanine site, *SLC6A4* solute carrier family 6 (neurotransmitter transporter, serotonin), member 4, *LEPR* leptin receptor, *GHSR1a* growth hormone secretagogue receptor 1a, *OXTR* oxytocin receptor, *LEP* leptin, *DNAm* methylation of deoxyribonucleic acid, *TNXB* tenascin XB, *HDAC4* histone deacetylase 4, *IGF2* insulin-like growth factor 2, *DRD2* dopamine receptor D2, *DRD4* dopamine receptor D4, *BDNF* brain derived neurotrophic factor, *POMC* proopiomelanocortin, *SLC6A3/DAT* dopamine transporter, *CNR1/CB1* cannabinoid receptor1, *ANP* atrial natriuretic peptide, *SNCA* synuclein alpha, *HERP* homocysteine-induced endoplasmatic reticulum protein, *EWAS* epigenome-wide association study, *NR1H3* nuclear receptor subfamily 1 group H member 3 (human), *MZ* monozygotic twins.

### Global DNA methylation measurements

Overall, four studies considered global DNA methylation patterns, which means that they measured the methylation levels of patients with AN and healthy controls only in parts of the genome related to the analysis method used (e.g., long interspersed nuclear elements-1 (LINE1) repetitive elements, methylation-sensitive restriction enzyme assay) [49, 58, 63, 64]. These publications provide information on global variations in methylation levels but cannot offer more specific information on disease-relevant methylation changes at specific genomic loci. This is also reflected in the obtained contradictory results, as two studies reported global hypomethylation [58, 63], one reported global hypermethylation [49] and another found no differences in global DNA methylation levels when comparing DNA from patients with AN and healthy controls [64]. These opposite effects could be related to the small sample size used, with a maximum of 32 patients.

### Epigenome-wide association studies (EWASs)

Five studies performed epigenome-wide methylation measurements in AN patients, quantified by high-throughput microarray techniques such as the Illumina Infinium EPIC Bead Chip Kit, assessing DNA methylation of nearly one million CpG sites in a single experiment simultaneously. In contrast to the determination of global DNA methylation patterns or candidate gene studies, EWASs focus on site-specific changes at many genomic loci that can be associated with disease-relevant genes. The first EWAS was performed by Booij et al. in 2015 and compared epigenome-wide methylation levels in DNA obtained from leukocytes extracted from blood between 29 women suffering from AN and 15 age-matched healthy controls [49]. This research group identified 14 hypermethylated CpG sites in the genome of patients with AN that were associated with 11 genes (*PRDM16, HDAC4, TNXB, FTSJD2, PXDNL, DLGAP2, FAM83A, NR1H3, DDX10, ARHGAP1, PIWIL1*) involved in, e.g., histone acetylation, thermogenesis, lipid transport or organization of synapses and neuronal cell signaling. They also associated illness chronicity with altered methylation levels involving gene pathways related to anxiety, immunity, and functioning of the central nervous system [49, 50].

The two most recent and largest EWASs were conducted by the same working group, with the number of participants being increased in the subsequent study, but they also added a group of female patients who were recovered from AN for at least one year [54, 61]. The first EWAS from Steiger et al [61] detected 58 differentially methylated CpG sites corresponding to 55 genes related to metabolic and nutritional status (e.g., *PRKAG2* (lipid processing), *RPTOR* (glucose metabolism)), psychiatric status (e.g., serotonin receptors) or immune function [61]. Most of these altered CpG sites (91.4%) exhibited an increased methylation profile in females with active AN. Interestingly, they could not find any significant differences in DNA methylation patterns between remitted patients and healthy controls using a longitudinal study design, suggesting that epigenetic changes in actively ill patients might be reversible. Although epigenetic changes could thus be reversible, larger studies of longitudinal data from the same patients should be examined throughout the entire course of illness, including recovery, to draw this conclusion with certainty. In the present study from Steiger et al., which was the only group that performed a longitudinal EWAS design, follow-up methylation measurements were conducted for only 52 of the 75 included patients suffered from active AN. Nevertheless, they found that an increase in BMI generally corresponded with an increase in methylation-level changes in genes associated with lipid and glucose metabolism, immune and inflammatory processes, and olfaction. In contrast, a longer duration of AN was connected to lower methylation, suggesting that DNA methylation profiles responded to short- and long-term variations in the disease period, again indicating the promising ability of DNA methylation changes as potential biomarkers of illness status [61]. The second EWAS, from Steiger et al [54], is the largest EWAS available to date, with a sample size of 145 acutely ill patients, 49 patients remitted for at least one year and 64 controls without an eating disorder background, increasing the sample size from Steiger et al [54, 61]. As in their other two studies [49, 61], several genes nominally showed altered methylation patterns in the case‒control comparisons, e.g., *SYNJ2, PRKAG2, STAT3, CSGALNACT1, GATA2, NOD1, NEGR1, and NR1H3*. Three of these (*PRKAG2, NOD1*, and *NR1H3*) were also described in the two earlier publications by this working group, while the others were not replicated. However, Steiger et al [54] were able to hint at a potential reversibility of DNA methylation after recovery, confirming the previous statement of a potential biomarker for disease status.

Iranzo-Tatay et al. recently published an epigenome-wide methylation study based on comparisons between 7 monozygotic twin pairs discordant for AN (MZ-AN) and 7 healthy monozygotic twin pairs. Since monozygotic twins share 100% of their genetic material, the evaluation of the epigenome can be analyzed independently from genetic sequence variation or age effects. The obtained results were then validated with the methylation profiles of 7 non-family-related age-matched healthy controls and 7 individuals suffering from AN [47]. Changes in the DNA methylation of 9 CpGs in MZ-AN samples were associated with different genes that reflect metabolic properties (*UBAP2L, LMNA, PPP2R2C, SYNJ2, ZER1, CHST1, JAM3, TUBA1A, FCHO1*).

Kesselmeier et al. determined the epigenome methylation pattern of 47 women with AN, 47 lean females without AN (lean) and 100 population-based (POP) healthy female controls The two included control groups (lean and POP) were used to display different aspects of potential disease-specific methylation differences. A comparison of lean women with patients suffering from AN evaluated starvation vs. low body weight as a key clinical feature because lean women also have a relatively low BMI but do not restrict their food intake. Comparing DNA methylation profiles between POP and AN cases more generally examined the typical aspects of AN [45]. Fifty-one differentially methylated CpG sites between patients with AN and lean controls and 81 differently methylated CpGs between individuals with AN and the POP group were identified. Validation of these results with monozygotic twins discordant for AN revealed that approximately 67% (54 of 81) of the obtained altered CpG sites showed directionally consistent mean methylation differences. Interestingly, each of the two genes associated with altered methylation in AN, *TNXB* and *NR1H3*, were already described in previous EWASs [45, 49]. *NR1H3* is involved in lipid metabolic processes, inflammation, and energy homeostasis, while *TNXB* encodes an extracellular matrix protein and is associated with connective tissue disorders [50, 65].

### Candidate gene studies

Candidate genes are selected based on prior knowledge related to the disease under investigation and prior hypotheses. A total of 16 publications focused exclusively on candidate genes in AN while some candidate genes were additionally considered in four EWASs. However, altered methylation profiles in AN compared to healthy controls were identified for only ten genes. These genes are related to mood regulation and neurotransmitter release (serotonin, dopamine, alpha-synuclein) [57, 58, 66–68], social attachment (oxytocin) [62, 69], appetite regulation (leptin, ghrelin) [53, 68, 70], lipid metabolism and energy homeostasis (*NR1H3*) [45, 49, 54, 61], extracellular matrix organization (*TNXB*) [45, 49, 54, 61] and regulation of gene expression (*HDAC4*) [49, 71]. Intriguingly, all genes except for leptin and, more recently, serotonin showed an increased methylation level in patients with AN compared to healthy controls [53, 66]. The included candidate gene studies are summarized in detail in Table. 1C and Table 2.

**Table 2:** Overview of genes and corresponding methylation results in the included studies focusing exclusively on candidate genes or additionally considering candidate genes in EWASs.

Gene	Alteration of DNAm in AN compared to HC	References
*SLC6A4 (serotonin transporter)*	↑ ↓ ↔	He et al. [67] Franzago et al. [66]. Pjetri et al. [68]
*SLC6A3/DAT (dopamine transporter)*	↑	Frieling et al. [57]
*OXTR (oxytocin receptor)*	↑ ↑	Thaler et al. [62] Kim et al. [69]
*DRD2 (dopamine receptor)*	↑	Frieling et al. [57]
*LEP/LEPR (leptin/leptin receptor)*	↓ (adult patients) patients with lowest LEP DNAm (high leptin expression = no appetite and low food intake) were the patients that fully responded to therapy and fully recovered ↔ (adolescence) ↔ (mixed)	Neyazi et al. [53] (longitudinal) Batury et al. [70] Pjetri et al. [68]
*GHSR1a (ghrelin receptor)*	↑	Batury et al. [70]
*NR1H3*	↑	Steiger et al. [61]
*TNXB*	↑	Kesselmeier et al. [45]
*HDAC4*	↑	Booij et al. [49]
*SNCA*	↑	Frieling et al. [58]

Another publication from Boehm et al. investigated methylation alterations in AN in the serotonin transporter gene *SLC6A4* and conducted an epigenome-brain-behavior pathway test with resting-state functional connectivity (rsFC) records [72]. They found that for AN, a variation in DNA methylation profiles of the *SLC6A4* gene was associated with a modulation in the functioning of brain circuitry linked to emotional regulation. However, these results need to be replicated and verified in future studies, and corrections for multiple testing are necessary.

## Discussion

We present a systematic literature search on epigenetic alterations specific to AN determined via DNA methylation patterns, including 23 studies with a total of 1075 patients and 956 healthy controls (overlapping individuals were considered). The studies included assessment of global methylation, characterization of epigenome-wide methylation studies, and determination of the methylation pattern in promoter regions of AN-specific candidate genes. No publications to date could be found on histone modifications or non-coding RNAs in AN. By extending the search period by almost five years, it was possible to include 11 additional studies on AN compared to the previous systematic review, especially regarding the analysis of epigenetic changes on an epigenome-wide level (EWAS). In general, the results of global methylation measurements and candidate gene studies are inconclusive or even partly contradictory; however, EWAS findings are promising. The five included EWASs (three independent studies) showed a reversibility of epigenetic changes upon recovery, that hints to malnutrition as a potential cause while not excluding other origins that were addressed by treatment. A restoration of the patients’ epigenetic profile to a state similar to that of the controls, would provide hope for both the affected individuals and their caregivers. These changes could also be used as a potential biomarker for disease staging. Overlapping genes showing differentially methylated CpG sites in patients with AN compared to healthy controls could help elucidate the underlying mechanisms of the progression and chronification of AN. Recommendations for future studies include larger sample sizes, uniform methods of analysis, and a longitudinal study design to determine individual, epigenetic long-term effects.

### Global DNA methylation measurements

Global methylation measurements were the first epigenetic measurements analyzed in case-control studies of patients with AN. However, they rendered inconclusive and contradictory outcomes and were not examined in more recent studies. For the determination of global methylation changes, only defined regions of the genome were considered. Methylation-sensitive restriction enzymes were used to examine methylation alterations only in promoter regions, or LINE1 (long interspersed nuclear elements-1) repetitive elements were characterized. These sequences are CpG rich but capture only 17% of the genome and 70% of total methylation [64, 73]. Thus, analyzing global DNA methylation cannot capture the entire methylation pattern. Furthermore, the methods do not reflect the direction of changes occurring at disorder-relevant sites. To date, no associations with disease-relevant epigenetic changes in specific genes have been revealed [73]. In contrast to the epigenome-wide analyses mentioned below, global methylation measurements may give somewhat misleading results and are rightly discontinued based on today’s knowledge.

### Epigenome-wide association studies (EWASs)

EWASs use chip-based epigenome-wide-assays and are not based on a priori hypotheses pertaining to the underlying mechanisms. They thus represent the current most valuable investigation because they have the greatest power to reveal disease-specific epigenetic patterns of change. In the future, whole epigenome-wide sequencing data will also become available. Currently, there are five EWAS approaches (three independent) examining epigenetic alterations in AN. Three of these are based on a participant number continuously increased from the first to the last study [49, 54, 61]. The most recent and largest EWAS, conducted by that working group, included 145 patients with acute AN and a group of 49 female patients who were recovered from AN for at least one year [54]. Furthermore, the EWAS performed by Steiger et al. in 2019 had a longitudinal study design, measuring follow-up methylation data of 52 individual participants after short-term weight restoration [61]. To exclude individual region-specific epigenetic changes that are not disease-related, such as those induced by environmental factors, diet, or drug intake, longitudinal characterization is a promising option for decoding the epigenome. The third large EWAS with 47 patients with AN was conducted by Kesselmeier et al [45]. and included an additional control group with lean females who had a similar BMI to the patients but did not restrict their food intake [45]. An efficient strategy to detect AN-specific epigenetic alterations is the evaluation of monozygotic twins discordant for AN, as the epigenome can be analyzed independently from genetic sequence variation, age, education, or various other environmental influences. Unfortunately, the recruitment of identical twins with anorexia is extremely challenging, resulting in very small case numbers. Therefore, only one EWAS has been performed to date, determining the epigenetic profiles of seven discordant identical twin pairs [47].

The five EWASs performed thus far detected several nominally AN-associated, differentially methylated CpG sites that could be assigned to different genes, with some genes overlapping between the different studies. The hypermethylation at multiple CpG sites annotated to the *TNXB* gene, identified in the first EWAS for AN from Booij et al [49], was replicated in a recent paper and analyzed in candidate gene studies [45, 49]. *TNXB* is responsible for extracellular matrix organization and is associated with connective tissue disorders [61]. AN-associated concordances in methylation differences in two or more studies were found at genes responsible for communication between nerve cells (*SYNJ2*), lipid metabolism and energy homeostasis (*NR1H3*) or thermogenesis (*PRDM16*), which are all highly interesting for the investigation of epigenetic changes in AN. Furthermore, Steiger et al [54] detected hypermethylation in the *NR1H3* gene when comparing the methylation patterns of patients with AN and healthy controls in their EWAS [54].

In the latest EWAS from Steiger et al. [54], altered methylation levels were measured at CpG positions that could be assigned to the *NEGR1* gene, when comparing females with active AN to age-matched healthy controls [54]. *NEGR1* encodes a cell adhesion molecule of the immunoglobulin superfamily that is expressed in several regions of the brain and thus is important for its connectivity [74–77]. Through the formation of homophilic and heterophilic complexes on the cell surface and between adjacent cells, cell adhesion and neurite growth are regulated. Cell adhesion molecules are crucial during neuronal development for additional complex cellular processes such as migration or synapse formation, as they facilitate interactions between cells and their environment [74, 78]. NEGR1 was one of the first genes identified in GWASs for BMI, including a meta-analysis of 15 publications with more than 30,000 participants, and demonstrated compelling evidence for a relation with obesity in adults and children [75]. This association with obesity suggested that the alterations in methylation detected in the loci of *NEGR1* are related to BMI in general and may be important for weight regulation. Some genetic studies have already shown this relationship to BMI at different loci [79, 80].

Alterations in epigenetic profiles were also observed in regions of the *STAT3* gene for patients with AN compared to healthy controls or recovered patients [54]. *STAT3* has been associated with AN and the transmission of leptin signaling in previous studies using the activity-based anorexia (ABA) rat model [81]. The ABA animal model is widely utilized and well-established in the study of AN. This model mimics the main characteristics of AN, such as loss of body weight, increased physical activity, amenorrhea, and reductions in brain volume by combining food deprivation with access to a running wheel [82–84]. Leptin, primarily released by white adipose tissue, serves as the primary messenger conveying information about peripheral energy reserves to the hypothalamus [85]. The binding of leptin to its most extended receptor isoform (ObRb) induces the phosphorylation of the STAT3 gene which forms dimers and relocates from the cytoplasm to the nucleus, activating anorexigenic factors leading to a decrease in body weight [86, 87] Hypoleptinemia is typically observed in patients with AN and plays a pivotal role in initiating the body’s response to starvation by influencing various areas of the brain, including the reward system. Changes in CpG methylation levels in individuals with AN compared to healthy controls could affect expression of the *STAT3* gene and therefore the leptin signaling pathway, leading to impaired regulation of appetite and energy balance and thus to metabolic or eating disorders. The Hebebrand group successfully supplemented patients with AN off-label with human recombinant leptin (metreleptin), which led to a quick onset of beneficial effects on cognition, emotion, and behavior [88–91].

Furthermore, the current updated World Federation of Societies of Biological Psychiatry (WFSBP) 2023 treatment guidelines for pharmacological treatment of eating disorders provide evidence for the use of olanzapine in AN [92]. Olanzapine was able to significantly increase BMI in adolescent patients with AN [93]. This substance also exhibits epigenetic effects, as epigenetic histone modulations contribute to olanzapine-induced metabolic disturbances [94]. Similarly, the use of cannabinoids in the treatment of eating disorders could be beneficial, as they also show a significant increase in weight gain, including exposure-related epigenetic changes in the brain [92, 95]. These two examples illustrate, that the treatment of AN is often associated with epigenetic effects on the pharmacodynamics of the drugs and should therefore be taken into consideration. Epigenetic mechanisms also influence the pharmacokinetics of various medications (e.g. antidepressants such as selective serotonin reuptake inhibitors (SSRIs) or serotonin–norepinephrine reuptake inhibitors (SNRIs)) prescribed to patients with AN to treat comorbidities such as depression and could at least partly explain their wide-spread lack of effect during starvation. The investigation in animal and human studies focuses on how drugs can induce epigenetic changes and how these alterations, in turn, affect the pharmacological mechanism of the drug or the response of the organism itself [96, 97]. This becomes particularly crucial in the context of future research aiming to develop personalized treatment options based on individual epigenetic profiles.

Interestingly, both longitudinal EWASs found alterations in the methylation levels in patients with acute AN but not in remitted individuals, hinting at a normalization in epigenetic changes after remission [54, 61]. This could signify that epigenomic alterations are mostly state effects, potentially due to semistarvation during the disease. The reversibility of changes in DNA methylation profiles after rehabilitation is a promising sign for patients. Therefore, the epigenetic profile could also be used as a biomarker for the illness state and may lead to targets for treatment options and development of nutritional supplements during starvation, affecting gene activation and protein synthesis. Clinical therapies targeting the epigenome have already been approved and adopted for cancer. In addition, clinical trials testing DNA methylation-sensitive drug treatments for neurological and metabolic diseases are already being conducted [98].

### Candidate gene studies

In total, 16 studies focused exclusively on hypothesis-driven candidate gene studies while some candidate genes were additionally considered in four EWASs. Altered methylation patterns in patients with AN were identified for the following ten genes: *SLC6A4, SLC6A3/DAT, OXTR, DRD2, LEP/LEPR, NR1H3, GHSR1a, TNXB, HDAC4*, and *SNCA*. Interestingly, except for the leptin gene (*LEP*), which was identified in the only longitudinal candidate study [53], and recently the serotonin receptor gene (*SLC6A4*) [66], all other genes showed hypermethylation in patients with AN compared to healthy controls (Table 2). The serotonergic system has been proposed to have a distinct impact on the pathophysiology of AN, given its pivotal role in regulating appetite, satiety, and mood [38, 99]. It is even regarded as a trait marker for AN [100, 101]. Moreover, it is proposed that the distortion of body image, a fundamental symptom of AN, might be associated with disturbances in the serotonergic system, particularly concerning the serotonin transporter (5HTT) [102]. Another interesting gene appears to be the Type 1 cannabinoid receptor gene (Cnr1), which is part of the endocannabinoid system. A significant increase in DNA methylation and thus a notable downregulation of the gene expression in the hypothalamus and nucleus accumbens have only been observed in the ABA animal model. Nevertheless, these data require further animal and human studies on the regulation of Cnr1 as a potential target for the treatment of AN [103].

However, candidate gene studies have thus far produced inconclusive or even contradictory results for epigenetic changes in different genes in patients with AN compared to healthy controls (Table 1C) [66, 67], or the results could not be reproduced in subsequent studies [49, 71]. This could be due to the small sample sizes; furthermore, some of the participants studied were a mix of acutely ill and recovered patients, which can lead to heterogeneity in the obtained results. The recovery process after acute AN can significantly affect epigenetics, as seen in the two longitudinal EWASs above, due to dietary changes, weight normalization, or hormonal changes, making data more difficult to compare. A further disadvantage is the fact that candidate gene studies are hypothesis-driven, and certain genes are selected based on prior knowledge, while others are ignored. A large area of the genome thus remains unexamined.

### Limitations and future directions

A major limitation is the low reproducibility of the studies to date. Altered methylation patterns were associated with different genes in the epigenome of patients with AN compared to healthy controls in each study, and the findings barely overlap. The low congruence of the obtained results could be due to the small sample size often used or the different cohorts varying in age, illness duration, or disease severity. Furthermore, it is important to consider other confounding factors that may influence epigenetic association studies. These include, for example, environmental influences such as medication intake (e.g., antidepressants), smoking, diet composition or alcohol consumption, as well as age, gender, or blood and hormone composition. Since these factors can interact with the individual epigenetic profile and thus distort the disease-associated results, it is important to correct for them through statistical analyses. There is evidence that the composition of food can influence the individual epigenetic profile [104–107]. Longitudinal studies as well as expanded group sizes are necessary to overcome these problems. The low reproducibility of results in the different EWASs may also be related to a recently described criticism. Over the last ten years, nearly all EWASs used commercial methylation detection arrays manufactured by Illumina (predominantly the HM450 and subsequently the scaled-up EPIC850 array) for population studies of DNA methylation in peripheral blood [108–111]. The measurement of the chips used for detecting CpG methylation are too rough, as 95% of the included CpGs do not show appreciable methylation differences among people. This interindividual variance serves as the basis for statistical correlations, and without variance within a population, the detection of methylation quantitative trait loci (mQTLs) becomes impossible [108, 112, 113]. Gunasekara et al [108] therefore developed an unbiased screen for correlated regions of systemic (i.e., not tissue-specific) interindividual epigenetic variation (CoRSIVs) in the human genome [108, 114]. These systematic epigenetic variants are promising for disease prediction, and improving the coverage of CoRSIVs could advance the utility of the Illumina EPIC array for studying population epigenetics in the future.

Another issue is the tissue specificity of epigenetic mechanisms because of their important role during cellular differentiation. Although AN has both psychiatric and metabolic components [12], all studies to date have measured epigenetic changes in whole blood, saliva-derived DNA, or buccal cells, rather than in brain, adipose, or intestinal tissue. For future studies, it would be interesting to determine methylation levels in metabolically active tissue. Nevertheless, studying brain tissue from patients with AN is a major challenge because it would only be possible in postmortem samples, which are rarely available and time, cause of death, and delay until sampling also add further complexities to epigenetic analyses. Animal studies, however, can fill this gap in the near future.

In addition, almost all publications included patients with a typical course of disease, neglecting those showing atypical AN (AAN). To reveal truly disease-specific epigenetic changes not exclusively related to significantly low body weight (criterion for a diagnosis of typical AN), patients with atypical AN should be included.

Finally, all previous epigenetic studies focused exclusively on DNA methylation changes in AN. Consideration of other epigenetic mechanisms, such as histone modifications or non-coding RNAs, especially microRNAs, should also be examined in future research. In addition, the interaction between genetics and epigenetics is important to understand for a better interpretation of the obtained epigenetic results related to AN.

A limitation of the present work is the omission of registration to databases such as PROSPERO or OSF prior to submission in order to increase scientific transparency and integrity. We will consider this for future work in order to minimize reporting bias. Moreover, a review with systematic literature search always has methodological limitations as self-selected and predefined inclusion and exclusion criteria may lead to the omission of some studies that contain important and interesting results.

## Conclusion

Epigenetic research in the field of AN is still limited and does not yet allow for firm conclusions because of factors such as small sample sizes, cross-sectional study designs or inconsistent application of covariates. The preliminary results hint to substantial changes during acute AN which likely alter gene activation during acute starvation and could potentially influence the maintenance and chronicity of the disease, with a high degree of reversibility upon recovery. However, these conclusions await confirmation via large international collaborations that are needed to achieve a sample size that is sufficient for correcting for the multiple covariates (ideally at least illness state, medication, smoking, diet composition, alcohol consumption, age, sex) and use the latest EWAS-technology (including improving the coverage of CoRSIVs) to draw valid conclusions from the obtained results. Furthermore, homogeneity in the employed analysis methods across different research centers will allow for future quantitative meta-analyses.

In addition, other epigenetic mechanisms, such as modification of histone proteins or microRNAs in patients with AN, should be investigated to better understand their role in gene regulatory effects of epigenetic variations. Due to the recent promising EWAS results showing the reversibility of DNA methylation changes after recovery in patients with AN, epigenetics, specifically DNA methylation patterns, may serve as a potential biomarker for disease status or early diagnosis in the future.

Finally, longitudinal studies are urgently needed to assess the individual effects of environmental exposures on the genome and to help determine the basis for epigenetic disturbances observed in AN. Longitudinal investigations could also aid in understanding disease staging and recovery as well as differentiating between state and trait effects. They can provide suggestions for disease-relevant biological pathways, which could serve as predictors for illness progression. They could also lead to at least two strategies for developing personalized disease-targeted therapeutic interventions. First, epigenetic research might help us identify the genes themselves that are relevant for the underlying pathophysiology of AN. Interesting genes for neuropsychiatric disorders such as AN could include neurotransmitters receptor genes (serotonin receptor, BDNF), for which both genetic and epigenetic mechanisms should be investigated by multiple research groups. To draw causal conclusions and subsequently be able to derive classical interventions for affected patients, animal studies are crucial. Secondly, a deeper understanding of involved epigenetic mechanisms could also be used directly for personalized epigenome-targeted therapeutic interventions such as those already being studied and clinically tested in cancer, neurological and metabolic diseases, all together giving new hope also to patients with AN.
